# Dystrophin in the Neonatal and Adult Rat Intestine

**DOI:** 10.3390/life11111155

**Published:** 2021-10-29

**Authors:** Judith M. Lionarons, Govert Hoogland, Rutger J. Slegers, Hellen Steinbusch, Sandra M. H. Claessen, Johan S. H. Vles

**Affiliations:** 1 Department of Neurology, Maastricht University Medical Center, 6229 HX Maastricht, The Netherlands; 2 School for Mental Health and Neuroscience, Maastricht University, 6229 ER Maastricht, The Netherlands; r.slegers@student.maastrichtuniversity.nl (R.J.S.); he.steinbusch@maastrichtuniversity.nl (H.S.); sandra.claessen@maastrichtuniversity.nl (S.M.H.C.); jsh.vles@mumc.nl (J.S.H.V.); 3 Department of Neurosurgery, Maastricht University Medical Center, 6229 HX Maastricht, The Netherlands

**Keywords:** dystrophinopathy, aging, peripheral nervous system, rat, intestine, development

## Abstract

Background: Gastrointestinal (GI) complaints are frequently noted in aging dystrophinopathy patients, yet their underlying molecular mechanisms are largely unknown. As dystrophin protein isoform 71 (Dp71) is particularly implicated in the development of smooth muscle cells, we evaluated its distribution in the neonatal and adult rat intestine in this study. Methods: Dp71 expression levels were assessed in the proximal (duodenum, jejunum and ileum) and distal (caecum, colon and rectum) intestine by Western blotting and qPCR. In addition, the cellular distribution of total Dp was evaluated in the duodenum and colon by immunohistochemical colocalization studies with alpha-smooth muscle actin (aSMA), Hu RNA binding proteins C and D (HuC/HuD) for neurons and vimentin (VIM) for interstitial cells. Results: In neonatal and adult rats, the distal intestine expressed 2.5 times more Dp71 protein than the proximal part (*p* < 0.01). This regional difference was not observed in Dp71 mRNA. During both stages, Dp-immunoreactivity was predominant in the muscularis propria, where it co-localized with aSMA and HuC/HuD. Conclusions: In neonatal and adult rats, Dp71 was expressed highest in the distal intestine. Together with the observation that Dp may be expressed by myenteric neurons, this warrants a paradigm shift in the treatment of GI comorbidities.

## 1. Introduction

Genetic defects resulting in dysfunctional protein expression are responsible for different forms of muscular dystrophies. In striated muscle, several muscular dystrophy-associated proteins, such as the dystrophin protein (Dp), have been identified [1]. However, Dp has also been found present in smooth muscle (i.e., bladder and intestine) and non-muscular tissues (i.e., brain, retina and kidney [2]). In dystrophinopathies, exon deletions in the DMD gene generally result in a loss of the functional ‘full-length’ dystrophin isoform (Dp427). In addition, the DMD gene also has at least four internal promotors that generate shorter protein products of 260 kDa (Dp260), 140 kDa (Dp140), 116 kDa (Dp116) and 71 kDa (Dp71) [3,4]. Based on our recent studies on Dp in the bladder, Dp71 is the most prominently expressed isoform in bladder smooth muscle and nervous tissue [5]. In addition, Dp71, in particular, is implicated during development [6,7,8,9]. Therefore, it is hypothesized that the intestinal Dp71 expression plays a role in the gastrointestinal (GI) comorbidities (i.e., dysphagia, gastric hypomotility and constipation [10,11,12,13]) that are observed in aging patients affected by dystrophinopathies [14]. In the field of gastroenterology, GI comorbidities are a well-known phenomenon in other types of muscular dystrophies, such as myotonic dystrophy. In myotonic dystrophy, these comorbidities have not only been attributed to motility disorders caused by striated or smooth muscle damage, but also to neurological alterations in, for example, the myenteric plexus [15,16]. In dystrophinopathies, such as Duchenne muscular dystrophy (DMD), the exact pathophysiology of these comorbidities is largely unknown.

Several human and animal studies have observed multiple Dp isoforms in smooth muscle cells and myenteric neurons in proximal and distal GI regions [5,17,18,19,20,21]. In addition to what the current literature states, structural changes of the GI wall during development may also potentially be associated with alterations in the Dp71 expression [22,23]. Here, we aimed to gain a better understanding of the GI comorbidities typically observed during aging in dystrophinopathies. To achieve this, we evaluated cellular Dp distribution (immunohistochemistry) and Dp71 expression levels (Western blot and quantitative polymerase chain reaction [qPCR]) throughout the intestine (duodenum, jejunum, ileum, caecum, colon and rectum) of healthy neonatal and adult rats. 

## 2. Materials and Methods

### 2.1. Animals

Immediately after decapitation, six anatomical regions of the intestine (duodenum, jejunum, ileum, caecum, colon and rectum) and the femoris muscle of the biceps were dissected from naive, 24 h (neonatal; N = 6) and six-month-old (adult; N = 15) male Sprague Dawley rats and snap-frozen in liquid nitrogen. Subsequently, all tissue samples were stored at −80 °C until immunohistochemical, Western blot and qPCR analysis. 

### 2.2. Western Blot

As described previously [24], tissues from the six anatomical regions of the intestine from both age groups (N = 6 24 h old and N = 12 six-month-old rats) were homogenized in a lysis buffer (1 g tissue per 9 mL lysis buffer), containing 0.01 M PBS, 1% Igepal, 0.1% Triton X-100, 1 mm ethylene glycol tetraacetic acid (EGTA) and 1 mm ehtylenediaminetetraacetic acid (EDTA) [25], and using glass beads (Analytik Jena AG, Jena, Germany) with a SpeedMill PLUS (Analytik Jena AG, Jena, Germany). Next, protein concentrations of the homogenates were estimated by a Bradford protein assay using the bovine serum albumin calibration curve (BioRad, Hercules, CA, USA; [26]). 

Proteins (100 μg proteins per sample) were resolved by 10% polyacrylamide gel electrophoresis. Each sample, including a loading control (the same rat intestine sample for each Western blot), was run (4 h, 90 V, 4 °C) in duplicate and then transferred (16 h, 90 mA, 4 °C) to a Polyvinylidene fluoride membrane (Millipore, Burlington, MA, USA). After the transfer, membranes were subsequently incubated in the Odyssey^®^ blocking buffer (LI-COR, Homburg, Germany) for 1 h at RT, in a 1:200 diluted anti-Dp primary antibody (Ab15277, Abcam, Cambridge, UK), a 1:50 diluted anti-aSMA primary antibody (ready-to-use, Dako, Santa Clara, CA, USA) and a 1:2,000,000 diluted mouse anti-glyceraldehyde-3-phosphate dehydrogenase (GAPDH) primary antibody (Fitzgerald Industries International, Acton, MA, USA) for 16 h at 4 °C, followed by a 1:10,000 diluted donkey anti-mouse IRDye 680RD secondary antibody (LI-COR) for GAPDH and aSMA and a 1:10,000 diluted goat anti-rabbit IRDye 800CW (LI-COR) for Dp 1 h at RT. All antibodies were diluted in the Odyssey^®^ blocking buffer.

Immunoreactive protein bands were visualized by an Odyssey infrared imaging system (LI-COR; [27]) and quantified by a blinded observer (R.J.S.) using ImageJ software [25]. Optical densities were measured as previously described [24,26,28]. First, Dp71 expression levels were normalized to the optical density of the respective GAPDH immunoreactive band and then normalized to the GAPDH immunoreactive band of an internal validity marker of the same adult colonic tissue sample for all Western blots.

#### Western Blot Data Analysis

The six anatomical regions of the intestine of 24 h old (N = 6) and six-month-old rats (N = 12) were analyzed, resulting in a total of 36 tissue samples for the 24 h old rats and 72 tissue samples for the six-month-old rats. The expression level of each sample was calculated as the average of one duplicate. These values were then used to calculate a mean ± standard error of the mean (SEM) for both age groups. 

To assess potential differences between the investigated segments of the intestine, two groups were formed for analysis purposes: proximal segment (duodenum, jejunum and ileum; N = 18 for 24 h old rats and N = 36 for six-month-old rats) and distal segment (caecum, colon and rectum; N = 18 for 24 h old rats and N = 36 for six-month-old rats). The proximal and distal segments were compared within and between both age groups using independent sample t-tests. Differences were considered statistically significant at *p* values of <0.05. Statistical analysis was performed using SPSS Statistics^®^ software (IBM SPSS Software, Inc., Armonk, NY, USA). 

### 2.3. qPCR

Tissue from the six anatomical regions of the intestine from the 24 h old (N = 6) and six-month-old (N = 6) intestine samples was homogenized in lysis solution from a GenElute Mammalian Total RNA miniprep kit (RTN70-1KT, Sigma-Aldrich, Saint-Louis, MO, USA) using InnuSPEED lysis tubes containing steel beads (Analytik Jena AG) with a SpeedMill PLUS (Analytik Jena AG). Next, the total RNA was isolated, according to the manufacturer’s protocol. RNA purity and concentration were assessed by optical density using a NanoDrop ND-1000 spectrophotometer (Thermo Fisher Scientific, Waltham, MA, USA). For cDNA synthesis, a total RNA of 300 ng was first incubated for 5 min at 65 °C to reduce secondary structures, and then reverse-transcribed using a RevertAid cDNA kit (K1622, Thermo Fisher Scientific) in a 20 μL reaction volume containing 1x Reverse Transcription buffer (48 mM Tris-hydrochloride, 50 mM potassium chloride, 4 mM magnesium dichloride, 5 mM dithiothreitol and a pH of 8.3), 2 mM deoxynucleotide mixture, 20 mM Oligo(dT)18 primers, 20 U/μL RNase inhibitor and 200 U/μL reverse transcriptases. This was incubated for 60 min at 42 °C and then for 5 min at 72 °C. All cDNA samples were stored at −20 °C until qPCR analysis.

Dp71-specific primers (gene accession number XM_017601911.1) were designed using the database and the primer-BLAST^®^ tool from the National Center for Biotechnology information (Table 1; [29]). Reference genes hypoxanthine phosphoribosyl transferase (Hprt), beta actin (ActB) and TATA box binding protein (Tbp) were chosen based on previous literature (Table 1; [30,31]) and evaluated for expression stability using Normfinder [32]. 

First, PCR (see protocol in Appendix A) was performed using a pilot sample (N = 1 adult rat colon), followed by a standard agarose gel electrophoresis (Thermo Fisher Scientific) of PCR products to verify whether the right products were used.

qPCR was performed in optical 96-wells plates using a LightCycler^®^ 480 System (Roch Life Science, Indianapolis, IN, USA). qPCR was carried out in a 10 μL reaction volume containing 5 μL SensiMix™ SYBR^®^ No-ROX Kit (QT650-02, Bioline Meridian Life Science Inc., London, UK), a 0.4 μM forward and reverse primer (Table 1) and 6 ng cDNA dissolved in nuclease-free water. A pooled sample for each age group containing all six anatomical regions of the intestine was included to set up a concentration curve and to determine efficiency. A “no-template” control containing reaction mix without cDNA and a negative control containing nuclease-free water were included to test for possible contamination of assay reagents. Samples were run in duplicate. PCR conditions comprised a 10 min denaturation at 95 °C, followed by 40 cycles of 15 s at 95 °C, 15 s at 60 °C and 15 s at 72 °C. Each PCR program was followed by a general dissociation curve protocol to check for product specificity. The PCR efficiency of Dp71 and the reference genes was determined by a standard curve of cDNA samples, according to the minimum information for publication of quantitative real-time PCR experiments guidelines [33].

#### qPCR Data Analysis

RNA copy numbers were quantified using the comparative Δ cycle threshold (Ct) method as follows [34]. Raw quantification cycle (Cq) values were first transformed into quantities. The quantities of the reference genes were expressed relative to the sample with the highest quantity and then used as data input for Normfinder [32]. The Normfinder algorithm estimates not only the overall expression variation of the candidate reference genes but also the variation between and within sample subgroups (six anatomical regions of the intestine for two age groups = 12 sample subgroups). The output consists of stability values (S) based on both intra- and intergroup expression variation. The most stable reference gene has the lowest S value.

For Dp71, the raw Cq values of the most stable reference gene were subtracted from the raw Cq values of the Dp71 gene expression level (Cq Dp71 gene - Cq reference gene = ΔCt). The ΔCt were used to assess differences in proximal and distal segments of the intestine within and between age groups using independent sample t-tests. PCR efficiency was calculated by LightCycler^®^ 480 System software (Roche Life Science, Penzberg, Germany) using 10^(-1/slope)-1*100. All primer sets were considered efficient between 90–110%. Statistical analysis was performed using SPSS Statistics^®^ software (IBM SPSS Software, Inc., Armonk, NYC, USA). Differences were considered statistically significant at *p* values of < 0.05.

### 2.4. Immunohistochemistry

For immunohistochemistry, 10 μm transversal sections of tissue from the six anatomical regions of the intestine (duodenum, jejunum, ileum, caecum, colon and rectum) and the femoris muscle from the biceps of neonatal (N = 3) and adult (N = 3) rats were serially cut using a cryostat (Leica Microsystems, Wetzlar, Germany). The reason we used a smaller sample size for immunohistochemistry than for the previous experiments (Section 2.3 and Section 2.4) is due to the difference between the aims of the Western blot and qPCR experiments (obtaining quantitative data of total dystrophin protein and mRNA expression), and the immunohistological evaluation (obtaining an overview of the cellular distribution pattern). Standard hematoxylin-eosin (H&E; Merck, Darmstadt, Germany) staining was performed to evaluate the general histology and verify the presence of a mucosal-, submucosal-, muscular- (muscularis propria) and adventitial layer (serosa) in all six regions of the intestine from both neonatal and adult rats [35]. 

In order to compare regional and cellular Dp distribution to that in the literature, the two most frequently studied proximal (duodenum) and distal (colon) regions of the intestine from neonatal (N = 3) and adult (N = 3) rats were stained for Dp (polyclonal rabbit anti-Dp primary antibody, 1:200, Ab15277, Abcam, Cambridge, UK; [17,20]). After the blocking of nonspecific antibody binding with 10% goat serum (Millipore, Etobicoke, Ontario, Canada) diluted in 0.1 m of phosphate-buffered saline (PBS) containing 0.01% Triton X-100 for 1 h at room temperature (RT), sections were incubated with the primary antibody for 40 h at 4 °C. After two nights, the sections were rinsed three times with PBS for 10 min at RT and incubated with the secondary antibody (goat anti-rabbit Alexa Fluor^®^ 488, 1:400, Thermo Fisher Scientific, Eugene, OR, USA) for 2 h at RT. Sections were coverslipped in PBS containing 80% glycerol. As a positive control, the bicep femoris tissue was stained in the same manner. The anti-Dp antibody used is known to stain all Dp isoforms containing a C-terminal (Dp71, Dp116, Dp260 and Dp427), as previously described [5].

For co-localization immunohistochemistry, Dp was double-stained with alpha-smooth muscle actin (aSMA) as a marker of smooth muscle cells, Hu RNA binding protein C and D (HuC/HuD) as a marker of neurons and vimentin (VIM) as a marker of interstitial cells (monoclonal mouse anti-aSMA primary antibody, ready to use, Dako, Glostrup, Denmark; monoclonal mouse anti-HuC/HuD primary antibody, 1:200, A-21271, Thermo Fisher Scientific; monoclonal mouse anti-VIM, 1:1000, MU074-UC, BioGenex, Fremont, California). Sections were quickly fixed using ice-cold acetone (EMSURE^®^, Millipore) for 10 min. Then, the nonspecific antibody binding was blocked with 10% goat and donkey serum (Millipore) diluted in 0.1 m of PBS containing 0.01% Triton X-100 for 1 h at RT for co-localization studies with aSMA and VIM or 4% goat and donkey serum (Milipore) diluted in 0.1 m of PBS containing 0.5% Triton X-100 for 2 h at RT for HuC/HuD [36]. Next, sections were incubated with primary antibodies: anti-Dp together with (1) anti-aSMA, anti-HuC/HuD or anti-VIM for 40 h at 4°C. After two nights, the sections were rinsed three times with PBS for 10 min at RT and incubated with secondary antibodies for 2 h at RT. Goat anti-rabbit Alexa Fluor^®^ 488 secondary antibody (1:400, Thermo Fisher Scientific) was used for Dp together with donkey anti-mouse Alexa Fluor^®^ 594 secondary antibody (1:200, Thermo Fisher Scientific) for aSMA, HuC/HuD and VIM. Additionally, sections were stained with Hoechst (1:500; 15 min at RT; Sigma-Aldrich, Saint-Louis, MO, USA) to visualize cell nuclei and coverslipped in PBS containing 80% glycerol. As a negative control, sections were incubated without the primary antibody. 

Photo micrographic images were made using a BX51 microscope (Olympus, Tokyo, Japan) connected to a Qimaging EXIAqua (Bioimaging solutions, San Diego, CA, USA) camera that generated 16-bit TIFF files for single staining and a Stereo Investigator Confocal Spinning-Disk (SI-SD) system microscope (MBF Bioscience, Williston, VT, USA) connected to a modified Olympus BX51 fluorescence camera (Olympus, Tokyo, Japan) with controlling software (Stereo Investigator, MBF Bioscience) that generated 16-bit TIFF files for double staining. Images were taken with fluorophore-dependent exposure times that were determined by detection limits in the negative controls (minus primary antibody). Exposure times were filter-specific (Hoechst 50 ms, Texas Red^®^ 1000 ms and Fluorescein isothiocyanate 500 ms). 

## 3. Results

### 3.1. Dp71 and aSMA Protein Expression in the Neonatal and Adult Rat Intestine

**Figure 1:**
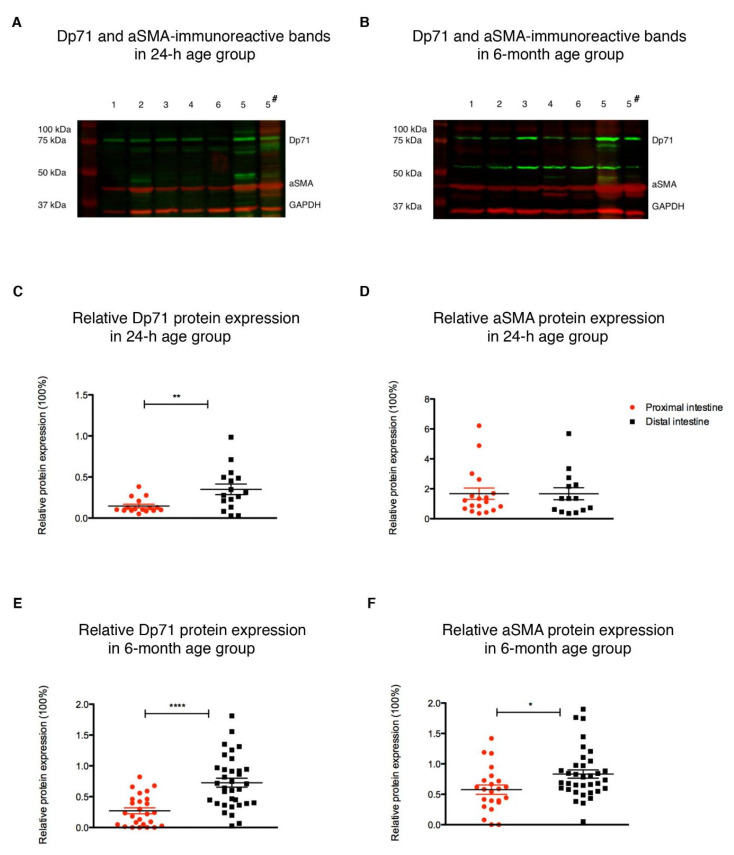
Dp71 and smooth muscle protein expression in the neonatal and adult rat intestine. Western blots showing dystrophin (Dp)-immunoreactive bands (green) at ~ 75 kDa (designated Dp71) and alpha-smooth muscle actin (aSMA)-immunoreactive bands at ~ 42 kDa (red) in all six investigated anatomical regions of the intestine (1, duodenum; 2, jejunum; 3, ileum; 4, caecum; 5, colon; 6, rectum) of 24 h old (**A**) and six-month-old (**B**) rats. Immunoreactive bands were quantified and expressed relative to their respective anti-glyceraldehyde-3-phosphate dehydrogenase (GAPDH) level (red; ~ 37 kDa) and an internal validity marker of the same adult colonic tissue sample for all blots (5#; right lane). Relative protein expression of Dp71 (**C**) and aSMA (**D**) in proximal (duodenum, jejunum and ileum) and distal (caecum, colon and rectum) segments of the 24 h old rats. Relative protein expression of Dp71 (**E**) and aSMA (**F**) in proximal and distal segments of the six-month-old rats. Data are presented as mean ± SEM of six 24 h and twelve six-month-old animals. * *p* < 0.05; ** *p* < 0.01; **** *p* < 0.0001. GAPDH = glyceraldehyde-3-phosphate dehydrogenase.

Western blots revealed that each of the six anatomical regions of the intestine contained a Dp-immunoreactive band (green) at approximately 75 kDa (Figure 1A,B), hereafter referred to as Dp71 [24]. Also, aSMA-immunoreactive bands (red) at approximately 42 kDa were observed in all samples (Figure 1A,B). Fourteen data points were excluded from analysis because their value was >2 standard deviations (SD) from the mean: a Dp71-immunoreactive band in three (N = 1 duodenum, N = 2 rectum) and an aSMA-immunoreactive band in four (N = 3 colon, N = 1 rectum) 24 h old rat samples, and a Dp71-immunoreactive band in two (jejunum and caecum) and an aSMA-immunoreactive band in five (N = 1 duodenum, N = 3 jejunum, N = 1 caecum) six-month-old rat samples. Due to the relatively small sample size, two groups were formed: the proximal segment (duodenum, jejunum and ileum) and the distal segment (caecum, colon and rectum) of the intestine for analysis purposes.

Within the 24 h old age group, Dp71 expression in the distal segment was about 2.5 times higher than that in the proximal segment (*p* = 0.005, Figure 1C). In this age group, aSMA expression in the proximal segment was comparable with the distal segment (n.s., Figure 1D). Within the six-month-old age group, the expressions of both Dp71 and aSMA were higher in the distal segment than in the proximal segment (*p* = 0.0001 and *p* = 0.010, respectively; Figure 1E,F). 

Between age groups, the expression of Dp71 throughout the intestine was comparable (n.s.) and the aSMA expression was higher in the six-month-old rats compared with the 24 h old rats (*p* = 0.010). 

### 3.2. Gene Expression of Dp71 in the Neonatal and Adult Rat Intestine

**Figure A1:**
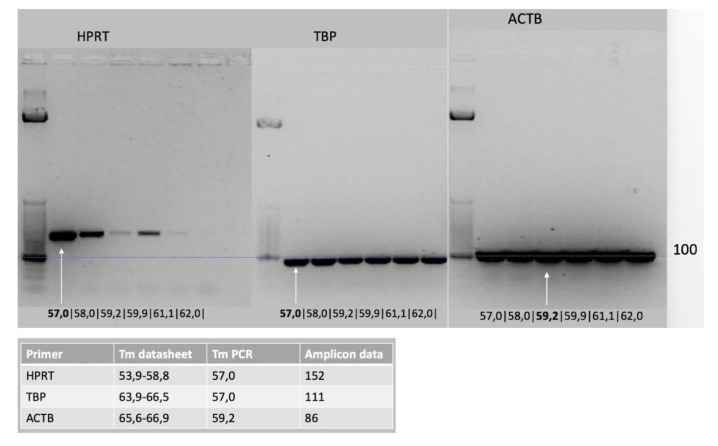
Reference gene PCR product in adult rat intestine pilot sample on agarose gel.

**Figure A2:**
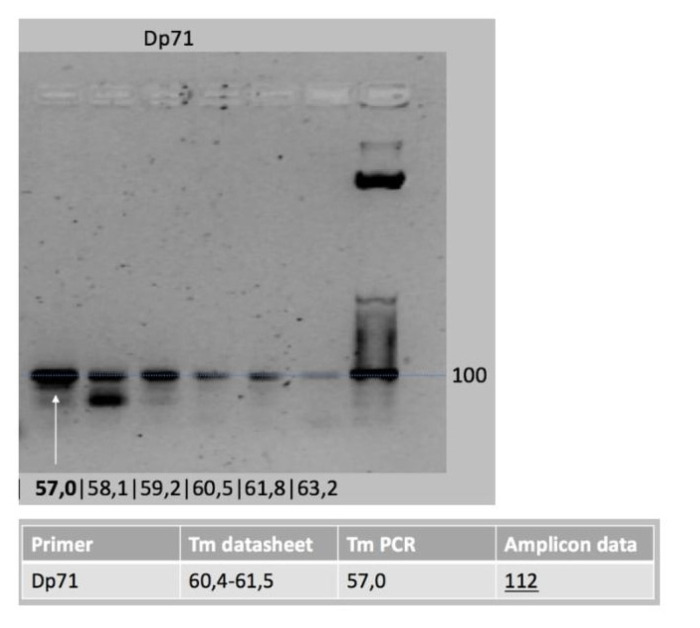
Dp71 gene PCR product in adult rat intestine pilot sample on agarose gel.

Prior PCR verification analysis confirmed that suitable products had been used for qPCR experiments (Figure A1 and Figure A2). For validation of candidate reference genes Hprt, ActB and Tbp, Normfinder was used to determine their overall expression variation and their variation between sample subgroups. This analysis ascertained that the order of stability was: Hprt (S = 0.567) > Tbp (S = 2.343) > ActB (S = 4.330), indicating that Hprt was the most stably expressed gene since it had the lowest S value. Even though the PCR efficiency of these genes ranged from 93.5 to 103.3%, indicating that all three were efficient, Hprt was used for qPCR analysis. 

**Figure 2:**
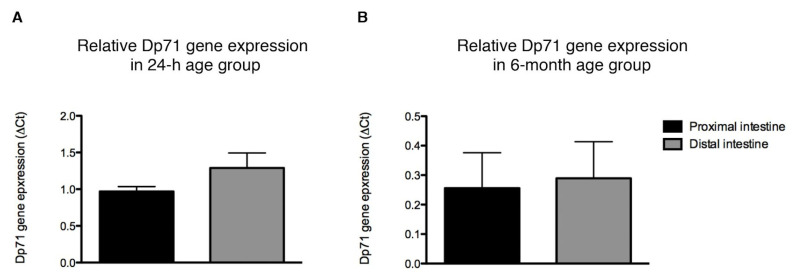
Dp71 gene expression in the neonatal and adult rat intestine. Relative gene expression of Dp71 in proximal (duodenum, jejunum and ileum) and distal (caecum, colon and rectum) segments of the intestine of 24 h old (**A**) and six-month-old (**B**) rats. All Dp71 *Cq* (quantification cycle) levels were expressed relative to their respective hypoxanthine phosphoribosyl transferase *(Hprt) Cq* levels (ΔCt = delta cycle threshold). Data are presented as mean ± SEM.

Within the 24 h old rats, Dp71 gene expression in the proximal segment did not differ from that in the distal segment of the intestine (n.s., Figure 2A). A similar observation was made in six-month-old rats (n.s., Figure 2B).

Between age groups, however, Dp71 gene expression throughout the intestine was about four times higher in the six-month-old rats compared with the 24 h old rats (*p* = 0.001).

### 3.3. Histological and Cellular Dystrophin Distribution in the Rat Intestine

**Figure 3:**
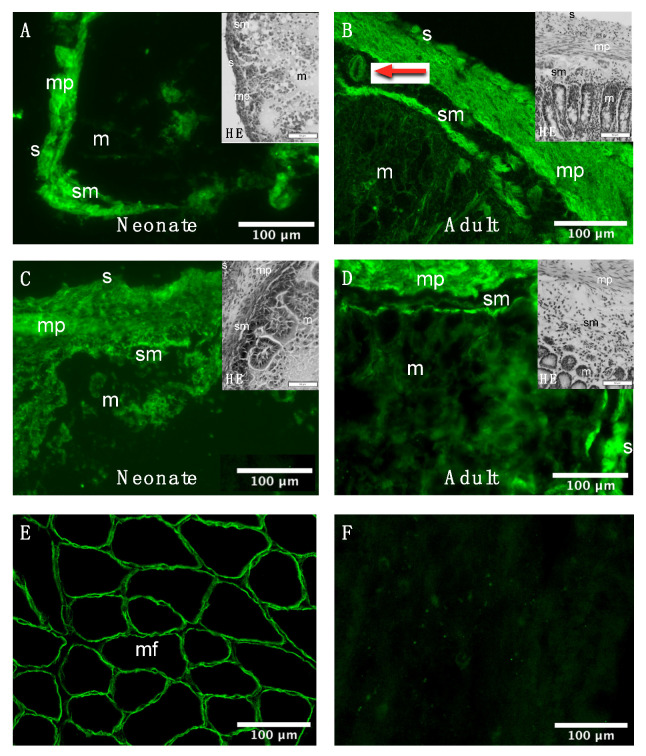
Dystrophin immunoreactivity in the neonatal and adult rat intestine. Dystrophin (Dp) immunoreactivity in neonatal (**A**) and adult (**B**) duodenum and neonatal (**C**) and adult (**D**) colon, 10 µm transverse section, ×200 magnified. In all samples, Dp is predominantly expressed in the *muscularis propria.* Other nearby anatomical regions, such as the serosa, show Dp-immunoreactivity to a lesser extent, similar to Vannucchi et al. [18]. The staining pattern furthermore suggests Dp expression in a blood vessel in the submucosa (**B**; red arrow). As a reference for anatomical histology, Hematoxylin and eosin (H&E) staining were added to the main figures, ×200 magnified, at a scale of 50 μm. See Appendix C for the original H&E staining of all six anatomical regions of the intestine. (**E**) Positive control: Dp immunoreactivity in the adult *bicep femoris* muscle, 10 µm transverse section, ×200 magnified. (**F**) Negative control: Minus primary anti-Dp antibody immunoreactivity in adult duodenum, 10 µm transverse section, ×200 magnified. m, mucosa; mf, myofiber; mp, *muscularis propria*; s, serosa; sm, submucosa.

**Figure A3:**
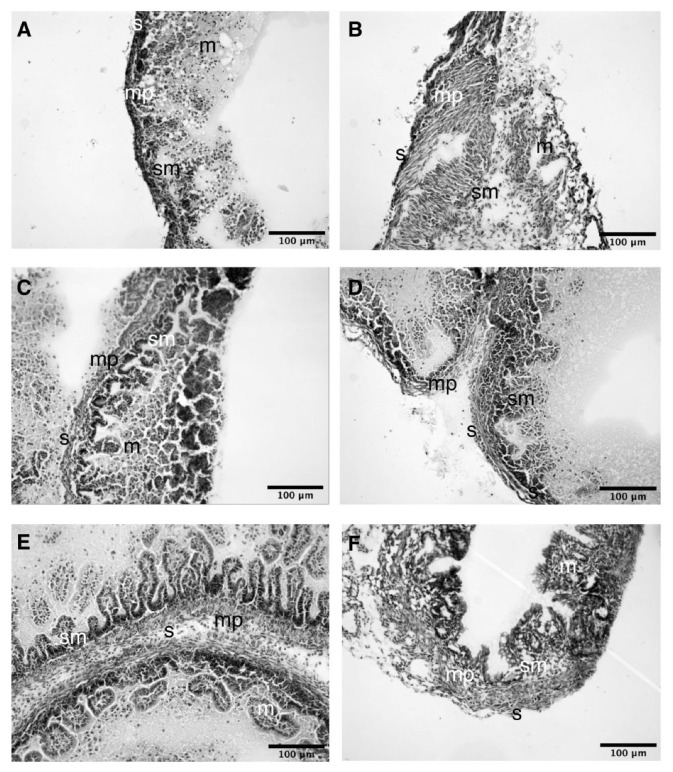
Histology of neonatal rat intestine. Hematoxylin and eosin staining of a 24 h old rat intestine (duodenum (**A**), jejunum (**B**), ileum (**C**), caecum (**D**), colon (**E**) and rectum (**F**)), 10-μm transverse section, ×100 magnified. m, mucosa; mp, *muscularis propria*; s, serosa; sm, submucosa.

**Figure A4:**
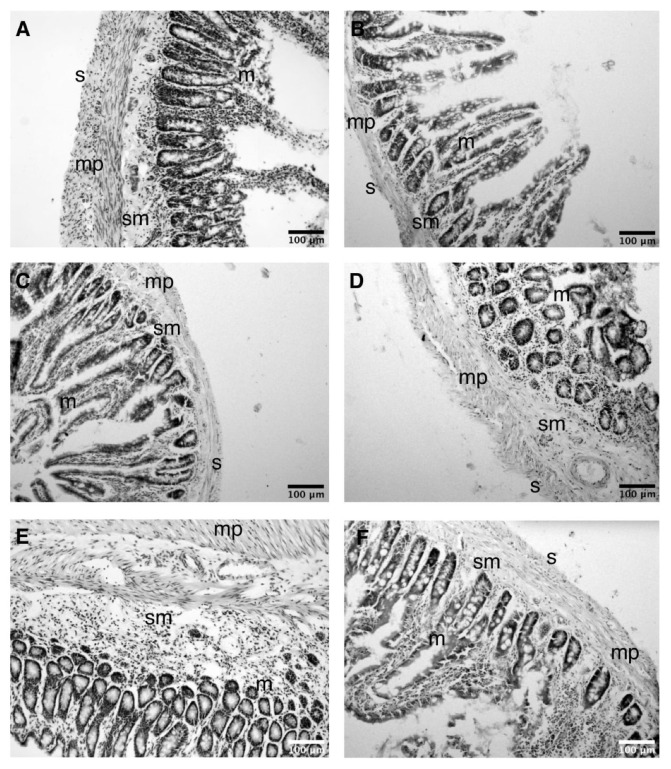
Histology of adult rat intestine. Hematoxylin and eosin staining of a six-month-old rat intestine (duodenum (**A**), jejunum (**B**), ileum (**C**), caecum (**D**), colon (**E**) and rectum (**F**)), 10-μm transverse section, ×100 magnified. m, mucosa; mp, *muscularis propria*; s, serosa; sm, submucosa.

The histochemical H&E staining of the adult rat intestine permitted the identification of the mucosal-, submucosal-, muscular- (muscularis propria) and adventitial layer (serosa) in all six anatomical regions of the intestine from neonatal (Figure A3) and adult rats (Figure A4). As these regions share this histological architecture [35], the histological and cellular distribution of Dp was evaluated in one proximal (i.e., duodenum) and one distal (i.e., colon) region. Dp-immunoreactivity predominantly appeared in the muscularis propria in both intestinal regions at both ages (Figure 3A–D). Additionally, other nearby anatomical regions such as the serosa appeared to show Dp-immunoreactivity to a lesser extent. Dp immunoreactivity was also observed in the submucosa, suggesting expression in blood vessels (red arrow Figure 3B). Controls included the bicep femoris muscle, showing Dp-immunoreactivity of myofibers in the sarcolemma (Figure 3E), and adult duodenum stained without a Dp antibody, showing no detectable signal at the same exposure time (Figure 3F).

**Figure 4:**
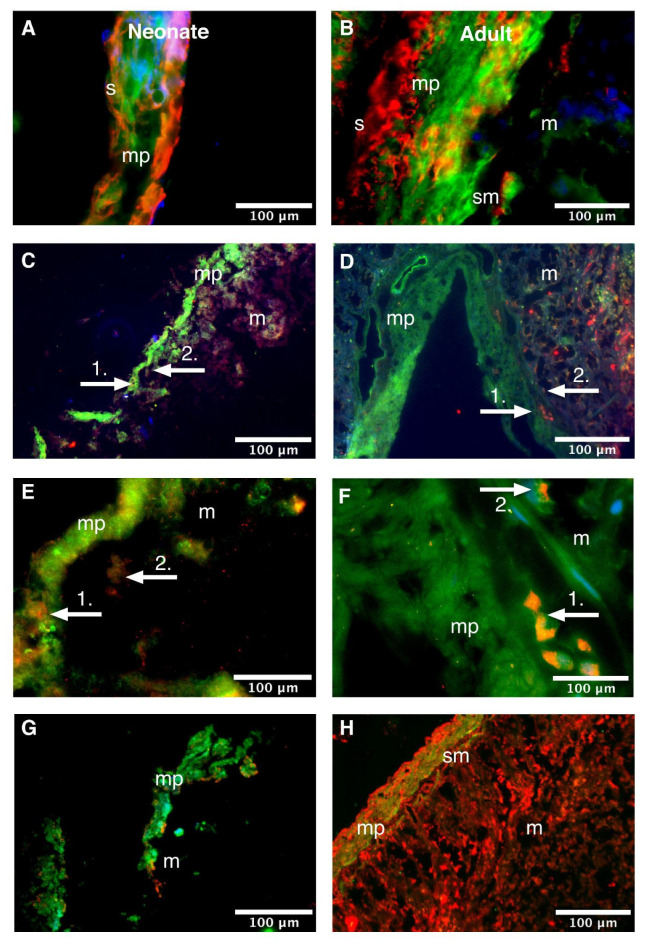
Cellular distribution of dystrophin in the neonatal and adult rat duodenum. Neonatal (**A**) and adult (**B**) duodenum stained for dystrophin (Dp; green), alpha-smooth muscle actin (aSMA; red) and Hoechst (blue), 10 µm transverse sections, ×600 magnified. Dp co-localized with aSMA in smooth muscle cells. Neonatal (**C**,**E**) and adult (**D**,**F**) duodenum stained for Dp (green), pan-neuronal marker Hu RNA binding protein C and D (HuC/HuD; red) and Hoechst (blue), 10 µm transverse sections, (**C**,**D**): x100 magnified, (**E**,**F**): ×400 magnified. HuC/HuD-immunoreactive neurons co-localized with Dp-immunoreactive cells in the *muscularis propria* (white arrows 1.) of both neonatal (**C**,**E**) and adult rats (**D**,**F**). At both ages, there appeared HuC/HuD-immunoreactive neurons in the (sub)mucosa (white arrows 2. in (**C**,**E**) [neonatal] and (**D**,**F**) [adult]) that did not co-localize with Dp. Neonatal (**G**) and adult (**H**) duodenum stained for Dp (green) and interstitial cell marker vimentin (red), 10 µm transverse sections, ×100 magnified. No co-localization of Dp and vimentin was observed. m, mucosa; mp, *muscularis propria*; s, serosa; sm, submucosa.

**Figure 5:**
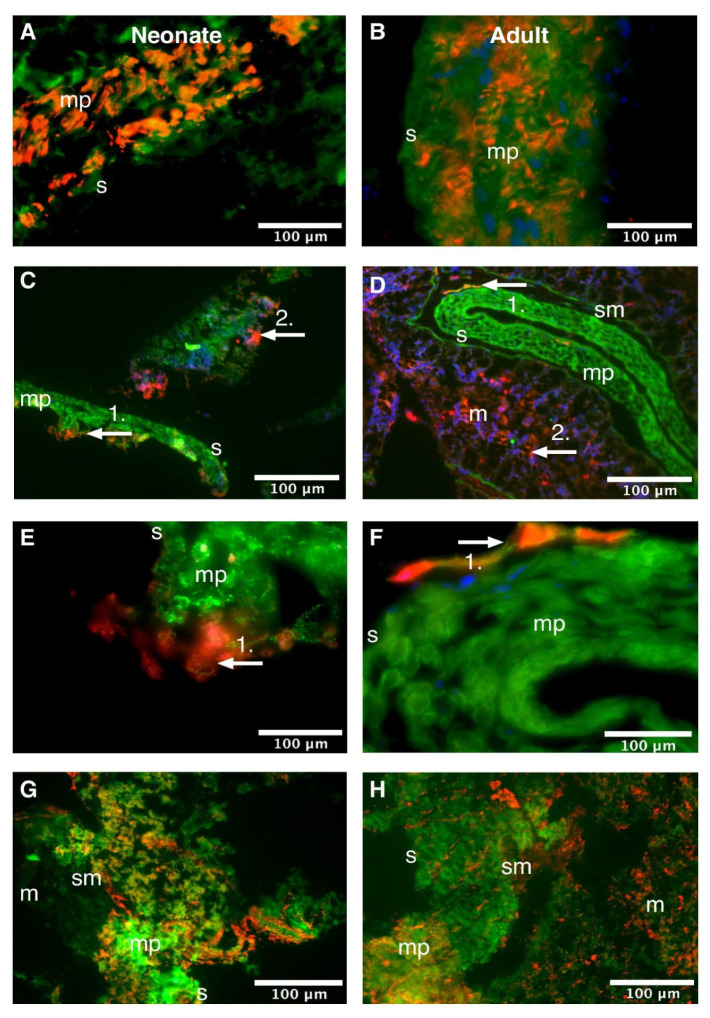
Cellular distribution of dystrophin in the neonatal and adult rat colon. Neonatal (**A**) and adult (**B**) colon stained for dystrophin (Dp; green), alpha-smooth muscle actin (aSMA; red) and Hoechst (blue), 10 µm transverse sections, ×600 magnified. Dp double labelled with aSMA in smooth muscle cells. Neonatal (**C**,**E**) and adult (**D**,**F**) colon stained for Dp (green), pan-neuronal marker Hu RNA binding protein C and D (HuC/HuD; red) and Hoechst (blue), 10 µm transverse sections, (**C**,**D**): ×100 magnified, (**E**,**F**): ×400 magnified. Dp-immunoreactive cells and fibers were found adjacent to HuC/HuD-immunoreactive neurons (white arrows 1.) in the *muscularis propria* of neonatal (**C**,**E**) and adult rats (**D**,**F**). At both ages, there appeared HuC/HuD-immunoreactive neurons in the mucosa (white arrows 2. in (**C**,**E**) [neonatal] and (**D**,**F**) [adult]) that did not co-localize with Dp. Neonatal (**G**) and adult (**H**) colon stained for Dp (green) and interstitial cell marker vimentin (red), 10 µm transverse sections, ×100 magnified. No co-localization of Dp and vimentin was observed. m, mucosa; mp, *muscularis propria*; s, serosa; sm, submucosa.

**Figure A5:**
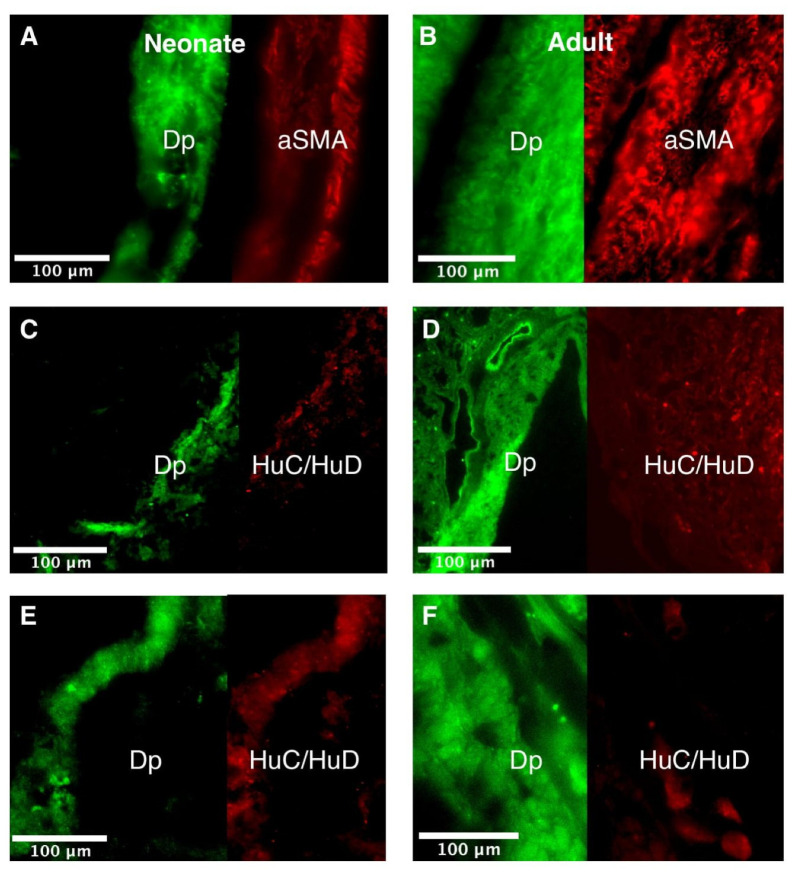
Cellular distribution of dystrophin in neonatal and adult rat duodenum. Channels (Fluorescein isothiocyanate and Texas Red^®^) were split for all immunohistochemical co-localization studies of the duodenum. Dystrophin (Dp; green) and smooth muscle marker alpha-smooth muscle actin (aSMA; red) in neonatal (**A**) and adult (**B**) duodenum, 10 µm transverse sections, ×600 magnified. Dp (green) and pan-neuronal marker Hu RNA binding protein C and D (HuC/HuD; red) in neonatal (**C**,**E**) and adult (**D**,**F**) duodenum, 10 µm transverse sections, ×100 magnified (**C**,**D**) and ×400 magnified (**E**,**F**).

**Figure A6:**
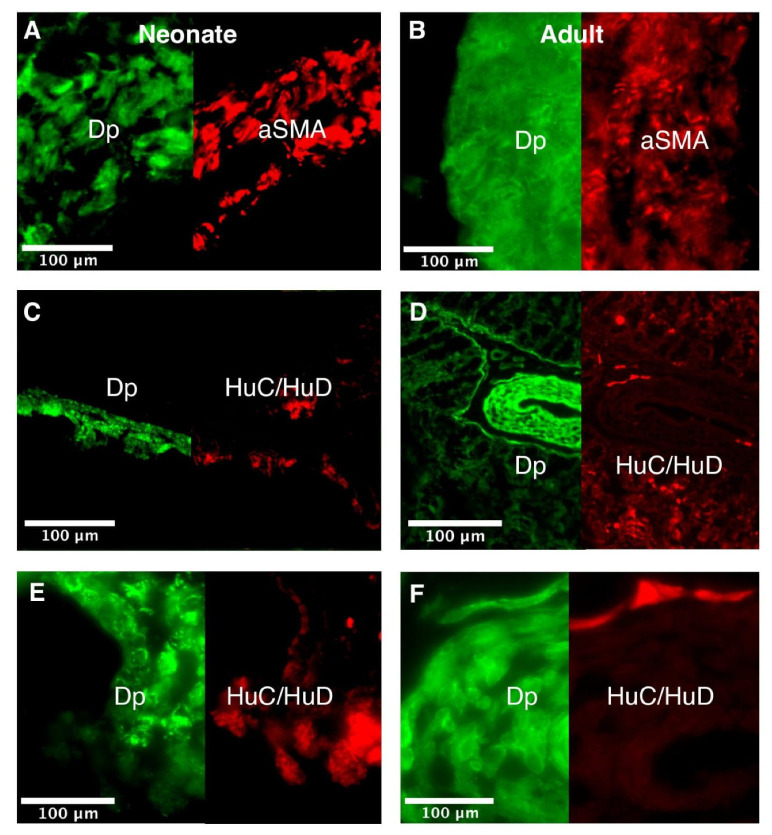
Cellular distribution of dystrophin in neonatal and adult rat colon. Channels (Fluorescein isothiocyanate and Texas Red^®^) were split for all immunohistochemical co-localization studies of the colon. Dystrophin (Dp; green) and smooth muscle marker alpha-smooth muscle actin (aSMA; red) in neonatal (**A**) and adult (**B**) colon, 10 µm transverse sections, ×600 magnified. Dp (green) and pan-neuronal marker Hu RNA binding protein C and D (HuC/HuD; red) in neonatal (**C**,**E**) and adult (**D**,**F**), 10 µm transverse sections, ×100 magnified (**C**,**D**) and ×400 magnified (**E**,**F**).

Immunofluorescent co-localization studies are presented as an overlay in Figure 4 (duodenum) and Figure 5 (colon), and as individual channels in Figure A5 (duodenum) and Figure A6 (colon).

Evaluation of the cellular distribution of Dp in the duodenum of neonatal rats showed that it co-localized with aSMA in the muscularis propria (Figure 4A). In addition, HuC/HuD-immunoreactive myenteric neurons were observed as isolated clusters adjacent to Dp-immunoreactive cells in the muscularis propria (white arrows 1 in Figure 4C,E and Figure A5C,E). Additionally, there were HuC/HuD-immunoreactive neurons in the (sub)mucosa that did not co-express Dp (white arrows 2 in Figure 4C,E). Finally, Dp did not co-localize with VIM in the neonatal duodenum (Figure 4G). Similar expression patterns were seen in the adult duodenum (Figure 4B,D,F,H, respectively).

The cellular distribution of Dp in the colon was comparable to that in the duodenum. The colon of neonatal rats also showed Dp-immunoreactivity that: 1. co-localized with aSMA in the muscularis propria (Figure 5A); 2. was expressed adjacent to HuC/HuD immunoreactive cells in the muscularis propria (Figure 5C); 3. did not co-localize with HuC/HuD immunoreactive cells in the (sub)mucosa (Figure 5E); and 4. did not co-localize with VIM (Figure 5G). Similar expression patterns were seen in the adult colon (Figure 5B,D,F,H, respectively).

## 4. Discussion

To gain a better understanding of the GI comorbidities in aging patients affected by dystrophinopathies, we evaluated histological and cellular Dp distribution and Dp71 expression throughout the neonatal and adult rat intestine. The main findings of this study are that: (1) the Dp71 protein expression was equally expressed throughout the intestine in both ages and was 2.5 times higher in the distal than in the proximal part of the intestine; (2) aSMA was 1. 5 times higher in the distal than in the proximal part of the intestine, yet this difference was only observed in adult rats; (3) Dp71 mRNA was four times higher in the adult than in the neonatal intestine—the proximal and distal part of the intestine, however, expressed equal amounts of Dp71 mRNA at both ages; and (4) Dp immunoreactivity was found in smooth muscle cells and in myenteric neurons in all evaluated specimen, i.e., the duodenum and colon of neonatal and adult animals.

### 4.1. Dp71 and aSMA Expression in Neonatal and Adult Rats

Interestingly, the expected aSMA upregulation we observed in the distal segment of the adult compared to the neonatal intestine was irrespective of the Dp71 expression pattern, which was equally higher in the distal segment of the intestine at both ages. A recent study on histological characteristics during the development of the colon and rectum found that the thickness of the smooth muscular layer increased with age, whereas the number of ganglion cells did not differ between neonatal and adult rats [37]. Analogously, this might lean towards a neurogenic role for Dp71 in the intestine during development that reaches beyond the support of solely smooth muscle functioning. In previous research on the developmental role of Dp71, high activity of the Dp71 promotor often seemed to be associated with morphogenic events and, in some tissues, this activity greatly increased towards birth [7]. Although it is well established in experimental studies with mice that the enteric nervous system (ENS) plays a critical role in the control of GI motility patterns in mature animals, the role of the developing ENS in motility patterns in both the duodenum and colon of neonatal animals is unclear [38]. As there is no reference data on intestinal Dp development available in the current literature, the explanation of higher Dp71 expression in the distal intestine compared to the proximal intestine of neonatal rats remains elusive and represents a major task for future research in postnatal ENS development in general. The large intestine (distal segment) is mainly involved in the water and electrolyte absorption of indigestible food material to form and propel feces towards the rectum for elimination, for which strong mass contractions of intestinal smooth muscle are necessary [39]. Consequently, the presence of Dp in intestinal smooth muscle may also partly explain the higher levels of Dp71 and smooth muscle in the large intestine of mature animals, even though intestinal contents strongly differ between mature and neonatal animals because of the different food intake. The mechanism involved in propulsion appears to match the consistency of the intestinal content in each anatomical region of the adult intestine, which suggests that the propulsive mechanism will match the consistency of the intestinal contents during development [40]. 

Although no differences in Dp71 gene expression were found between the distal and proximal segment of the intestine in both age groups, we found a four times higher Dp71 mRNA expression throughout the intestine in adult rats compared to neonatal ones. Prior PCR verification analysis confirmed that the same Dp71 product was used for qPCR as for Western blot (Figure A1). A possible explanation of the discrepancy between Western blot and qPCR results may be that the Dp protein and messenger RNAs have a different turnover [41]. Also, mRNAs are less stable than proteins [42,43]. Notably, structural proteins such as Dp have a much longer lifespan than regulatory proteins [42]. Further investigation of the rates and abundances of the Dp71 gene may thus help to gain insights into physiology that is relevant to Dp in various tissues.

The gap of knowledge regarding the effect that dystrophinopathy may have on GI motility and consequent clinical symptoms warrants further research. This may include translating preclinical findings to clinical studies. In future preclinical studies, models that specifically target smooth muscle or neuronal dystrophin may address the role of dystrophin with respect to the functional differences between the proximal and distal intestines. Future clinical studies may aim at diagnosing these comorbidities earlier in the disease course, for instance by histopathological examination. This may help to differentiate between a possible neurogenic or myogenic basis of GI comorbidities and herewith may improve the understanding of the pathophysiology of involvement of the GI tract in neurological conditions, such as DMD. This may eventually lead to a paradigm shift in the treatment of GI comorbidities.

### 4.2. Cellular Distribution of Dystrophin in the Rat Intestine

These co-localization studies show that Dp is expressed in clusters that lay adjacent to the HuC/HuD immunopositive myenteric neurons in the muscular layer. This neuronal network appears even more present in adult rats than in neonatal ones, which corroborates with the observation that the three-dimensional architecture of the ENS changes during maturation from a densely packed plexus to an expanded, fiber-rich network [44]. It is currently of particular interest whether age-related changes in the intestinal microbiome are the main factor causing functional and structural changes in the ENS. This hypothesis already exists for other neurological diseases, such as Parkinson’s disease [45]. Future studies targeting Dp may also take this into account, as a similar mechanism could affect dystrophinopathies. Moreover, as a next step in targeting Dp for treatment, it is crucial to investigate pathological tissues.

In addition, the observation that Dp was expressed by intestinal smooth muscle cells is in line with previous studies [17,46]. Though the myenteric plexus is primarily involved in the control of the smooth muscle motor pattern (peristalsis), the role of smooth muscle Dp in GI functioning is not fully understood. Dp-deficient Golden Retriever dogs and mdx mice that miss the Dp link between the dystrophin–glycoprotein complex and the actin cytoskeleton, show an altered phenotype and functioning of alveolar smooth muscles [47]. Dp may play a similar role in GI musculature.

Other nearby anatomical regions, such as the serosa, also appeared to show Dp-immunoreactivity to a lesser extent. As immunofluorescence preparations were unfixed cryosections, recognition of the anatomical regions was lost over time due to disintegration of the tissue, which made it difficult to differentiate between regions. The reason we used unfixed cryosection is of a technical nature, i.e., autofluorescence issues after formalin fixation, as is commonly seen in intestinal tissue. The autofluorescence disappeared when using unfixed fresh frozen tissue, in accordance with Crockett et al. [17]. Reporting on this technical issue is of importance, as it may reduce the duration of the protocol optimization period for other researchers, which may aid future research. 

The immunohistochemical data also showed expression of Dp in blood vessels in the submucosa, which may be arterioles entering the mucosa. Since Dp has previously been described to support actin dynamics that regulate the contractile differentiation of vascular smooth muscle cells in mice, a similar role for it might be present in the arterioles of the GI tract [19]. As these cellular expression patterns were obtained by using an antibody that recognized all Dp isoforms, the extent to which the quantified Dp71 levels reflect these different cell types remains to be established. This information, together with future experiments showing a loss-of-function of a specific Dp isoform, may contribute to a better understanding of GI comorbidities and will aid the development of targeted therapies.

## 5. Conclusions

Dp is expressed by smooth muscle cells and is found in the myenteric neurons of neonatal and adult rats. Regionally, Dp71 was expressed predominantly in the distal intestine, irrespective of age. The finding of not only regional differential myogenic, but also of neurogenic dystrophin expression between the proximal and distal intestines underscores that GI comorbidities cannot solely be ascribed to muscle weakness and immobility. This may lead to a clinical paradigm shift asking for a different therapeutic approach targeting nervous control, such as (sacral) neuromodulation. Whether age-related changes in the intestinal microbiome are mainly responsible for functional and structural changes in the ENS remains to be studied.

## Figures and Tables

**Table 1:** Reference and Dp71 gene-specific primers. ActB, beta actin; Hprt, hypoxanthine phosphoribosyl transferase; Tbp, TATA box binding protein; Dp71, dystrophin protein isoform 71 kDa; F, forward primer; R, reverse primer; bp, base pairs. Primer sequences of reference genes were based on literature [30,31] and the primer sequence of Dp71 was designed using the database (gene accession number XM_017601911.1) and the primer-BLAST^®^ tool from the National Center for Biotechnology information database [31]. All primers were obtained from Sigma Aldrich (Saint-Louis, MO, USA).

Gene Symbol	Gene Function	Primer Sequence (5′ → 3′)	Amplicon Length (bp)
*ActB*	Cytoskeletal structural protein	F: AGAGGGAAATCGTGCGTGAC R: CGATAGTGATGACCTGACCGT	86
*Hprt*	Purinergic enzyme	F: AAGAGCTACTGTAATGACCA R: GTATCCAACACTTCGAGG	152
*Tbp*	General transcription factor	F: TAATCCCAAGCGGTTTGCTG R: TTCTTCACTCTTGGCTCCTGTG	111
*Dp71*	Dystrophin protein isoform 71 kDa	F: ACAAGCACAGGGTTAGAAGAA R: AGGCTTCCTACATTGTGTCCT	112

## Data Availability

The data presented in this study are available on request from the corresponding author. The data are not publicly available by choice of the research team.

## Abbreviations

ActB	beta actin
aSMA	alpha-smooth muscle actin
CNS	central nervous system
DGC	dystrophin-glycoprotein complex
DMD	Duchenne muscular dystrophy
Dp	dystrophin protein
ENS	enteric nervous system
GAPDH	anti-glyceraldehyde-3-phosphate dehydrogenase
GI	gastrointestinal
Hprt	hypoxanthine phosphoribosyl transferase
HuC/HuD	Hu RNA binding protein C and D
PBS	Phosphate buffered saline
Tbp	TATA box binding protein
VIM	vimentin

## Appendix A. Polymerase Chain Reaction Protocol

After denaturation for 10 min at 95 °C, cDNA was amplified using a three-step program (35 cycles of 30 s at 94 °C, 60 s at 72 °C and 10 min at 72 °C) performed with the LightCycler^®^ 480 System (Roch Life Science, Indianapolis, IN, USA). Specificity of amplification was verified by the melt curve analysis.

## Appendix B

The left side of the picture shows a base pair (bp) marker for each reference primer with 100 bp as a reference line: ActB, beta actin; Hprt, hypoxanthine phosphoribosyl transferase; Tbp, TATA box binding protein. Below the picture and in the table, the melting temperatures (Tm) used for the PCR analysis and the Tm and amplicon length (bp) of the reference primers are visible. Based on gel electrophoresis, we confirmed that the right products with a similar amplicon length were used.

The left side of the picture shows a base pair (bp) marker for Dp71 primers with 100 bp as a reference line. Below the picture and in the table are the melting temperatures (Tm) used for the PCR analysis and the Tm and amplicon length (bp) of Dp71 primers. Based on gel electrophoresis, we confirmed that the right products with a similar amplicon length were used.
